# Anicteric Leptospirosis-Associated Meningitis in a Tropical Urban Environment, Brazil

**DOI:** 10.3201/eid2609.191001

**Published:** 2020-09

**Authors:** Scott A. Nabity, Guilherme C. Araújo, José E. Hagan, Alcinéia O. Damião, Mitermayer G. Reis, Albert I. Ko, Guilherme S. Ribeiro

**Affiliations:** Massachusetts General Hospital, Boston, Massachusetts, USA (S.A. Nabity);; Instituto Gonçalo Moniz, Salvador, Brazil (G.C. Araújo, A.O. Damião, M.G. Reis, A.I. Ko, G.S. Ribeiro);; Yale University, New Haven, Connecticut, USA (J.E. Hagan, M.G. Reis, A.I. Ko);; Universidade Federal da Bahia, Salvador, Brazil (M.G. Reis, G.S. Ribeiro)

**Keywords:** leptospirosis, leptospiral meningitis, neuroleptospirosis, aseptic meningitis, viral meningitis, case series, viruses, meningitis/encephalitis, Brazil

## Abstract

While studying aseptic meningitis in Salvador, Brazil, we diagnosed anicteric leptospirosis in 1.7% (5/295) of patients hospitalized for aseptic meningitis. Leptospirosis-associated meningitis patients had lower mean cerebrospinal fluid cell counts and protein than other-cause aseptic meningitis (p<0.05). Clinicians must consider leptospirosis-associated meningitis in appropriate clinical-epidemiologic contexts.

The clinical signs of leptospirosis can vary widely, which complicates timely diagnosis and targeted therapy. Aseptic meningitis associated with *Leptospira* infection, hereafter termed leptospirosis-associated meningitis (LAM), has been well described (*1**–**3*). However, clinicians diagnose atypical forms of leptospirosis less frequently, particularly in the absence of classic signs (i.e., renal insufficiency or hepatitis). Detection requires a high level of clinical suspicion, and even then imperfect diagnostics for leptospirosis limit timely confirmation.

Leptospirosis causes »1 million illnesses annually; this figure does not include LAM (*4*). Despite the high global burden of leptospirosis and early knowledge that anicteric LAM is underappreciated (*1*), few existing studies delineate the proportion of aseptic meningitis caused by *Leptospira* sp. infection in settings in which it is endemic. We performed surveillance for aseptic meningitis in an area of high transmission of urban leptospirosis. Hospital Couto Maia, the Oswaldo Cruz Foundation, and Yale University provided ethics approval for this study.

## The Study

We conducted the study at a public referral hospital for infectious diseases in Salvador, Brazil, April 18–October 18, 2012, during a period of seasonal increased risk for severe leptospirosis such as Weil’s disease (*5*) and severe pulmonary hemorrhagic syndrome (*6*). We enrolled consecutive patients >5 years of age who had a diagnosis of aseptic meningitis, defined by clinical meningitis (fever with severe headache or meningismus); nonturbid, nonpurulent cerebrospinal fluid (CSF) containing 10–2,000 cells/m^3^, protein <150 mg/dL, glucose >40 mg/dL; and negative results for bacterial meningitis on Gram stain, culture isolation, and latex agglutination tests. From the patients with aseptic meningitis, we aimed to select those most likely to elicit clinical suspicion for leptospirosis; a selection requirement was >1 epidemiologic risk factor for classic leptospirosis in the 30 days before symptom onset for leptospirosis testing. Risk factors were contact with floodwater, sewer water, or mud; rats at home or work; and residence or employment in a high-risk environment (i.e., slum community or animal farm) (7). We confirmed LAM by either *Leptospira* blood culture on EMJH media using 150 µL inoculum, or by reactive microagglutination test (MAT) by >1 of the following criteria: >4-fold acute-convalescent titer rise; seroconversion (undetectable acute-phase titer and convalescent-phase titer >1:200); or sample titer >1:800.

The public hospital received regional referrals for lumbar puncture and CSF analysis for suspected meningitis as standard practice; CSF showing >10 cells/mm^3^ required inpatient observation. At enrollment, we obtained demographic, epidemiologic, and clinical data from patients. We followed the patients during hospitalization to ascertain outcomes. We compared clinical characteristics between leptospirosis-confirmed and unconfirmed patients >15 years of age to avoid age confounding. Comparisons were made using Fisher exact test (2-tailed, α = 0.05) and Wilcoxon rank-sum test (2-tailed exact, α = 0.05).

We identified 295 patients with aseptic meningitis >5 years of age at admission. Of these, 22 (7.5%) had >1 epidemiologic risk factor for classic leptospirosis and therefore met criteria for leptospirosis diagnostic testing. Five (23%, 95% CI 7%–44%) of the 22 had confirmed LAM. Among all 295 patients, noting that 22 of these were tested for leptospirosis, the proportion LAM-confirmed was 1.7% (95% CI 0.5%–3.9%). MAT titers were highest against *L. interrogans* serogroup Canicola for 2 patients and *L. interrogans* serogroup Icterohaemorrhagiae for 2 others with paired serum specimens (Table 1). A fifth patient confirmed by an acute titer >1:800 had mixed highest titers against *L. interrogans* serogroup Icterohaemorrhagiae and *L. kirschineri* serogroup Cynopteri. None had a positive hemoculture for *Leptospira*, and none had clinical jaundice, which we defined as anicteric.

**Table 1:** Characteristics of patients with leptospirosis-associated meningitis, Salvador, Brazil*

Characteristic	Patient 1	Patient 2	Patient 3	Patient 4	Patient 5
Age, y/sex	24/M	18/F	38/M	42/F	32/M
Health history	Previously healthy	Crack cocaine and methamphetamine use; aseptic meningitis 8 months prior†	Previously healthy	Diabetes mellitus type II, obesity, and hypertension	Previously healthy
Risk factors	Rats present near home and exposed to both flood and sewer water	Rats present near home and exposed to sewer water	Rats present near home and exposed to flood water	Rats present near home and pet dog with outdoor exposure‡	Lived and worked on horse farm
Presenting symptoms	5 d of fever, severe headache, emesis, anorexia, malodorous urine, and intermittent dry cough with rare streaking hemoptysis§	7 d of fever, severe headache, emesis, anorexia, indigestion, diarrhea, nonproductive cough, and myalgia	8 d of fever, chills, severe headache with retroorbital pain, neck stiffness, photophobia, emesis, myalgia, and arthralgia¶	8 d of fever, severe headache, neck stiffness, myalgia, emesis, and abdominal pain	4 d of fever, severe headache, myalgia, and pharyngitis
No. healthcare encounters before diagnosis	2	0	2	1	1
Neurologic exam	Unremarkable	Unremarkable	Unremarkable	Nuchal rigidity	Unremarkable
CSF profile					
Nucleated cells, 10^6^/L	28	46	150	68	46
Predominant cell type	Monomorphic	Monomorphic	Polymorphic	Monomorphic	Monomorphic
Glucose, mg/dL	67	46	54	143#	77
Protein, g/L	46	35	41	32	23
Peripheral leukocytes with differential					
Total count, 10^3^/Ml	15.5	4.9	10.4	11.5	6.7
Neutrophils, %	85	33	62	84	76
Lymphocytes, %	6	45	30	11	9
Monocytes, %	5	2	1	4	8
Eosinophils, %	2	20	ND	1	3
Platelets, 10^3^/μL	108	243	336	268	119
Plasma chemistries					
Potassium, meq/L	5.2	4.2	4.1	ND	4.3
Creatinine, mg/dL	1.8	0.6	1.2	1.0	0.8
Urea, mg/dL	16	18	46	39	14
Bilirubin, direct, mg/dL	0.1	ND	ND	ND	ND
ALT, U/L	40	30	76	ND	25
Confirmation criteria	MAT seroconversion	MAT acute titer ≥1:800†	MAT seroconversion	MAT seroconversion	MAT seroconversion
Presumed serogroup	Icterohaemorrhagiae	Icterohaemorrhagiae and Cynopteri	Canicola	Canicola	Icterohaemorrhagiae
Other diagnostics (negative or within normal limits)	Rapid HIV; aerobic hemoculture; Rumpel-Leede test**	Rapid HIV; urinalysis	None	None	None
Bedside finger stick leptospirosis DPP result††	Negative	Negative	Negative	Positive	Positive
Venous whole blood leptospirosis DPP result‡‡	Positive	Positive	Negative	Positive	Positive
Antimicrobial treatment					
Regimen	Amoxicillin for 7 d	None	Ceftriaxone for 7 d	None	Ceftriaxone for 7 d
Day of illness started	Day 29 ‡		Day 8		Day 4

*ALT, alanine aminotransferase; CSF, cerebrospinal fluid; DPP, Dual Path Platform; MAT, microagglutination test; ND, not done. †No etiology was determined at prior hospitalization. The patient’s high acute sample MAT titer (1:800) is suggestive of acute leptospirosis, but may also represent either recurrent disease or recent prior infection. No convalescent serum sample was available for this patient. ‡The patient’s pet dog tested negative for leptospirosis by serum MAT and cultures of urine and blood. §This patient initially improved with 7 d of inpatient supportive management. However, the patient returned to the same hospital 3 d after discharge (day 15 of illness) reporting continued headache. The patient refused another CSF exam and returned home without treatment. On day 25 of illness, the patient returned with 2 d of renewed fever (39°C), headache, myalgia, emesis, and cough. Physicians suspected a viral illness and the patient returned home without antimicrobial therapy. On day 27 of illness, the patient returned to the same hospital with the additional report of malodorous, normochromic urine, at which time the patient presented the MAT results (resulted from the initial hospitalization) that confirmed the diagnosis of leptospirosis and the patient was prescribed amoxicillin on day 2 of re-admission. ¶Mild respiratory distress developed within the initial 24 h of admission and leptospirosis was included in the differential diagnosis on day 2 of hospitalization. #Blood glucose 298 mg/dL (CSF:blood ratio 0.48) **The Rumpel-Leede test is a clinical exam using a blood pressure cuff to screen for hemorrhagic manifestations of dengue. ††DPP was performed immediately at bedside via ventral pad finger stick (1). ‡‡DPP using venous whole blood was collected in an ethylenediamine tetraacetic acid (EDTA) tube and processed within 2 h of collection.

No patient was suspected to have leptospirosis at initial evaluation. However, 1 (patient 3) was subsequently suspected to have leptospirosis within 24 hours of hospitalization when a successive clinician noted mild respiratory distress with bilateral infiltrates on chest radiograph. Three confirmed patients ultimately had antimicrobial drug treatment (Table 1); none required admission to the intensive care unit or died, and all left the hospital at neurologic baseline. LAM patients had lower CSF cell counts and protein than patients with meningitis from other causes (Table 2).

**Table 2:** Characteristics of patients with leptospirosis-associated and non-leptospirosis meningitis, Salvador, Brazil*

Characteristic	Confirmed leptospirosis-associated meningitis			Nonleptospirosis aseptic meningitis		
No. patients	No. (%) or mean ± SD (range)	No. patients†	No. (%) or mean ± SD (range)	p value
Age, y	5	30.8 ± 9.9 (18–42)		13	28.6 ± 13.4 (16–51)	0.746‡
No. days of symptoms before hospital care	5	6.4 ± 1.8 (2–8)		13	6.2 ± 5.3 (2–20)	0.922‡
Symptom profile§						
Emesis	5	4 (80.0)		13	8 (61.5)	0.615
Photophobia	5	1 (20.0)		13	0	0.278
Nuchal rigidity	5	1 (20.0)		13	5 (38.5)	0.615
Abdominal pain	5	2 (40.0)		13	2 (15.4)	0.533
Diarrhea	5	2 (40.0)		13	2 (15.4)	0.533
Myalgia/arthralgia	5	4 (80.0)		13	6 (46.2)	0.314
Total peripheral eukocytes,10^3^/mL	5	9.8 ± 4.2 (4.9–15.5)		13	9.2 ± 1.2 (5.2–20.4)	0.800‡
Platelets, 10^3^/μL	5	214.8 ± 98.6 (108–336)		13	262.8 ± 52.6 (178–350)	0.193‡
Plasma chemistries						
Potassium, meq/L	4	4.5 ± 0.5 (4.1–5.2)		11	4.3 ± 0.5 (3.6–5.2)	0.504‡
Creatinine, mg/dL	5	1.1 ± 0.5 (0.6–1.8)		12	1.1 ± 0.3 (0.5–1.6)	0.910‡
ALT, U/L	4	42.8 ± 23.0 (25–76)		10	33.6 ± 9.5 (12–49)	0.297‡
Cerebrospinal fluid profile						
Nucleated cells, 10^6^/L	5	67.6 ± 48.2 (28–150)		13	351.3 ± 402.6 (17–1500)	0.025¶
Glucose, mg/dL	4	61.0 ± 13.7 (46–77)#		12	60.9 ± 11.6 (44–84)	0.991‡
Protein, g/L	5	35.4 ± 8.8 (23–46)		12	66.3 ± 31.3 (34–141)	0.014¶

*ALT, alanine aminotransferase. †Analysis restricted to patients >15 y of age to avoid confounding of biochemical parameters by age. ‡2-sample *t*-test using pooled variance. §All patients had severe headache and none had a seizure. ¶Wilcoxon sum-rank nonparametric test. #Excludes 1 patient with type 2 diabetes who had a blood glucose of 298 and a cerebrospinal fluid glucose of 143.

## Conclusions

The overall frequency of anicteric LAM among aseptic meningitis patients appeared low, despite being measured during Salvador’s highest rainfall season. We increased diagnostic yield to 23% with elementary risk stratification for classic leptospirosis. Because this process was likely to miss LAM that did not meet our inclusion criteria for testing, we probably underestimated the true frequency of LAM.

Lower cell counts and CSF protein levels distinguished LAM from other causes of aseptic meningitis, although the small sample size of our study limits their reliability. CSF pleiocytosis accompanies most cases of leptospirosis, particularly during the secondary immune phase of classic disease (*2**,**3*), even without clinical meningeal signs. Although typical CSF chemistries for LAM are well known (i.e., slightly elevated cell count and protein; normal-low glucose) (*2**,**3*), only 1 previous report showed direct comparison between the CSF of anicteric LAM and nonleptospirosis aseptic meningitis (*8*). However, the report did not include epidemiologic information and the CSF data were presented in a way that limited statistical comparison.

Two of 5 cases of LAM had the highest MAT titer against serogroup Canicola, which is associated with canine reservoirs (*9*). Serogroup Icterohaemorrhagiae predominates for classic leptospirosis in this setting (»90%) and is typically associated with rat (*Rattus norvegicus*) reservoirs (*5*). Thus, our findings suggest that there may be notable differences in the epidemiology of LAM and of classic leptospirosis as well as differential pathogenic mechanisms among these serogroups (*10*). Various serogroups have been implicated in anicteric LAM; they include Icterohaemorrhagiae (*8*,*11**–**13*) and Canicola (*11*,*12*,*14*) from differing epidemiologic settings over time.

We selected our screening criteria to affirm leptospirosis risk based on our epidemiologic understanding of classical leptospirosis. Criteria less grounded in classical leptospirosis risk exposures in this setting (e.g., including exposure to dogs), may help stratify aseptic meningitis patients for diagnostic testing. Similarly, CSF analysis alone is unlikely to properly identify candidates for leptospirosis diagnostic investigation. We did not culture CSF for *Leptospira*, nor could we attempt PCR on CSF; these limitations may have reduced diagnostic yield.

We ascertained leptospirosis as the cause of meningitis in a setting of high endemic transmission of urban leptospirosis, where severe disease forms are a predominant clinical presentation, but little information has been obtained for aseptic meningitis. Our findings support the development and validation of risk stratification strategies for systematic assessments of aseptic meningitis aiming to detect LAM in regions in which leptospirosis occurs endemically or epidemically. Such strategies, perhaps expanded to include nonrodent animal exposures, would not only help to guide diagnostic work-up but may also direct early introduction of antimicrobial drugs in settings without laboratories capable of diagnosing leptospirosis. Until there is an effective approach to differentiate anicteric LAM from other-cause aseptic meningitis at clinical presentation in low-resource environments, clinicians must maintain a robust and enduring index of suspicion for leptospirosis when evaluating patients with aseptic meningitis.
